# Supplementary material for: TIcagrelor in Rotational Atherectomy to Reduce TROPonin Enhancement: The TIRATROP Study, A Randomized Controlled Trial
Source: J Clin Med. 2023 Feb 11;12(4):1445. doi: 10.3390/jcm12041445 (PMC9964861; doi:10.3390/jcm12041445)
Supplement: Supplementary file 1 [file jcm-12-01445-s001.zip › jcm-2205564-supplementary.pdf]

On line only supplementary Material S1 : Exclusion criteria

Acute coronary syndrome with ST-elevation

Lesion located on a coronary bypass

Coronary thrombus diagnosed by angiography

Coronary dissection diagnosed by angiography

Left ventricular ejection fraction lower than 30%

Contraindication to Ticagrelor or Clopidogrel, as listed in the Summary of Product Characteristics

Known hypersensitivity to the active substance or to the excipients

Active pathological bleeding

History of intracranial hemorrhage

Moderate to severe hepatic impairment

Co-administration with a strong CYP3A4 inhibitor (e.g. ketokonazole, claritrhomycin, nefazodone, ritonavir, and atazanavir)

Other conditions of increased risk of bleeding (congenital or acquired coagulation disorder, gastroduodenal bleeding within past 6 months, recent major trauma or surgery within past 30 days)

Concomitant use of fibrinolytics, oral anticoagulation, Gp IIb/IIIa inhibitors, non- steroidal anti-inflammatory drugs,

Significant anemia

Increased risk of bradycardia

History of severe asthma or severe Chronic Obstructive Pulmonary Disease

Uric acid nephropathy

Ischemic stroke within 7 days

Heredity galactose intolerance, Lapp lactase deficiency, or glucose-galactose malabsorption

Concomitant use of a strong CYP3A4 inducer

Concomitant use of CYP3A4 substrates with narrow therapeutic indices (e.g. cisaprid, ergot alkaloids), simvastatin at a dose greater than 40 mg/d

Concomitant use of Selective Serotonin Reuptake inhibitors

Concomitant use of digoxin without close clinical and laboratory monitoring

Contraindication to Aspirin use

Breast-feeding

Pregnancy

Adult under protective administration

Patient participating in another biomedical study.

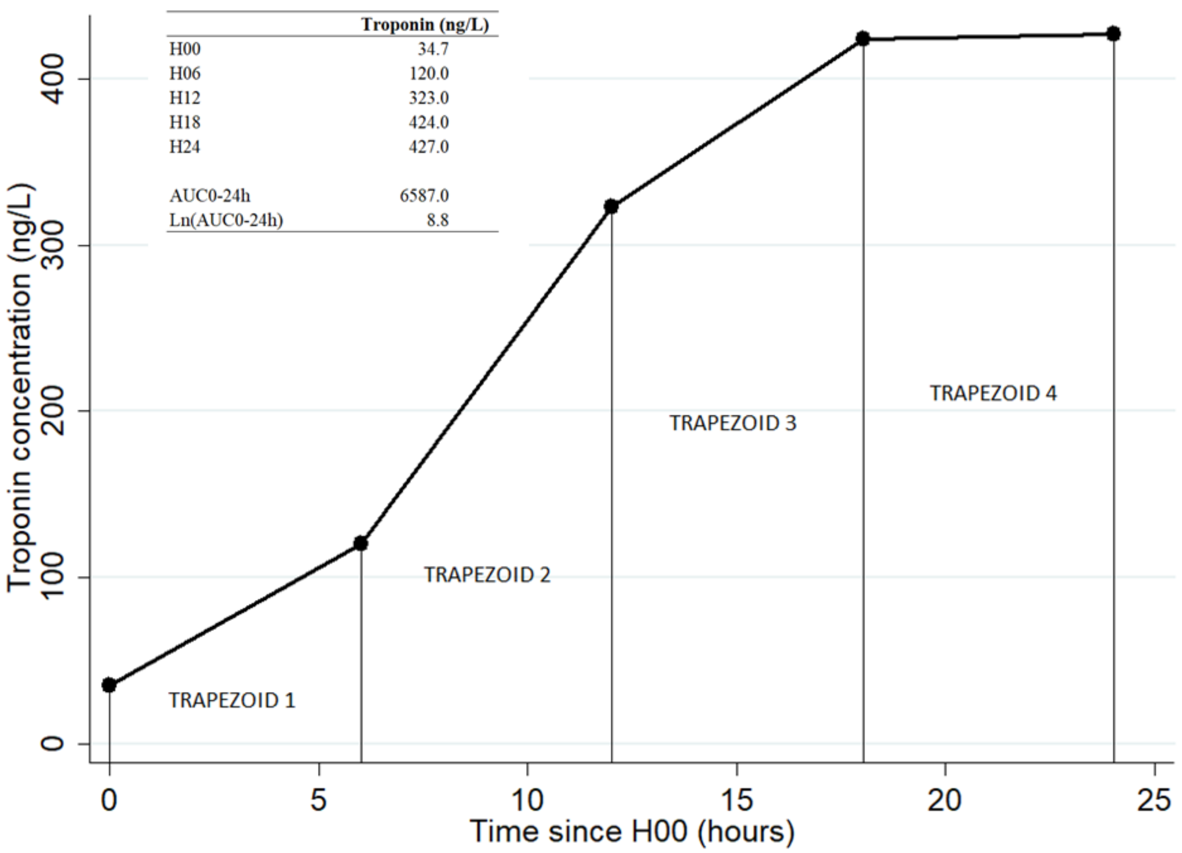

Online-only Supplementary Material S2: Illustration of post-procedural high sensitivity troponin T release measurement and aera under the curve calculation

AREA $_{\text{TRAPEZOID 1}}$ = 6 x (34.7+120)/2 = 464

AREA $_{\text{TRAPEZOID 2}}$ = 6 x (120+323)/2 = 1329

AREA $_{\text{TRAPEZOID 3}}$ = 6 x (323+424)/2 = 2241

AREA $_{\text{TRAPEZOID 4}}$ = 6 x (424+427)/2 = 2553

AUC 0-24h = 464+1329+2241+2553 = 6587

AUC: Area under the curve

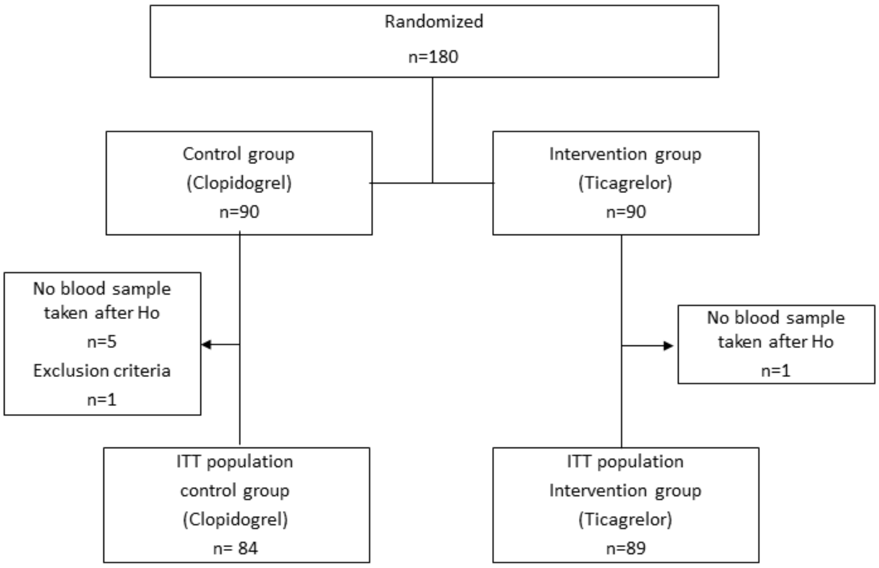

On line only supplementary Material S3: study flow chart

Online-only Supplementary Material S4: Factors associated with troponin elevation

(Ln(AUC24h)) – Univariate analysis

	n	Ln(AUC 24h)	p
Clinical status			< 0.001
Stable angina	151	7.6 +/- 1.0 *	
Post Acute ACS	22	9.1 +/- 1.1	
Gender			0.020
Male	132	7.7 +/- 1.1	
Female	41	8.1 +/- 1.3	
Age (years)	173 †	0.179	0.019
CARDIOVASCULAR RISK FACTORS			
Smoking			0.411
No	88	7.9 +/- 1.1	
Former smoker	62	7.7 +/- 1.2	
Current smoker	22	7.7 +/- 1.4	
Arterial hypertension			0.100
No	48	7.6 +/- 0.9	
Yes	125	7.9 +/- 1.2	
Hypercholesterolaemia			0.067
No	83	7.9 +/- 1.2	
Yes	89	7.6 +/- 1.0	
Type 2 Diabetes mellitus			0.506
No	112	7.7 +/- 1.2	

Yes	61	7.9 +/- 1.1	
INITIAL CLINICAL PARAMETERS			
BMI (kg/m²)	173	-0.036	0.640
Cardiac frequency (bpm)	172	0.106	0.167
Systolic Blood Pressure (mmHg)	171	0.073	0.345
Diastolic Blood Pressure (mmHg)	171	-0.092	0.229
LVEF (%)	121	-0.057	0.536
INITIAL BIOLOGICAL PARAMETERS			
Total cholesterol (mmol/L)	122	0.108	0.238
HDL cholesterol (mmol/L)	122	-0.010	0.910
LDL cholesterol (mmol/L)	122	0.082	0.369
Haemoglobin (g/100 mL)	166	-0.217	0.005
Platelets (thousands of cells)	164	-0.037	0.636
Glycaemia (mmol/l)	77	0.331	0.003
GFR (mL/min/1.73m2) ‡			0.004
≥ 60	111	7.6 +/- 1.1	
< 60	61	8.1 +/- 1.1	
C-Reactive protein (mg/L)	150	0.207	0.011
Leucocytes (cells)	168	-0.049	0.528
INITIAL CORONAROGRAPHY			
Left main			0.370
< 50%	109	7.7 +/- 1.1	

≥ 50%	63	7.9 +/- 1.2	
LAD			0.304
< 50%	14	7.5 +/- 1.1	
≥ 50%	159	7.8 +/- 1.1	
Cx			0.597
< 50%	57	7.7 +/- 1.2	
≥ 50%	116	7.8 +/- 1.2	
RCA			0.521
< 50%	33	7.9 +/- 1.3	
≥ 50%	140	7.8 +/- 1.1	
ROTATIONAL ATHERECTOMY PROCEDURE			
RA procedure for left main			0.938
No	140	7.8 +/- 1.2	
Yes	31	7.8 +/- 1.0	
RA procedure for LAD			0.066
No	83	7.6 +/- 1.0	
Yes	88	8.0 +/- 1.2	
RA procedure for Cx			0.173
No	146	7.7 +/- 1.1	
Yes	25	8.1 +/- 1.4	
RA procedure for RCA			0.498
No	109	7.8 +/- 1.2	
Yes	62	7.7 +/- 1.0	

Vascular access			0.235
Radial	124	7.7 +/- 1.1	
Femoral	23	8.0 +/- 1.3	
Sheath caliber (Fr)			0.170
6	135	7.8 +/- 1.1	
7	12	7.4 +/- 1.0	
Procedural success			0.716
Yes	169	7.8 +/- 1.1	
No	4	8.0 +/- 0.8	
Number of lesions treated with RA			< 0.001
1	123	7.6 +/- 1.1	
> 1	48	8.3 +/- 1.1	
Reference Vessel Diameter (mm) §	171	-0.2	0.009
Predilatation			0.308
No	11	8.1 +/- 1.0	
Yes	158	7.8 +/- 1.1	
PCI for another lesion during the same procedure			0.345
No	120	7.9 +/- 1.2	
Yes	51	7.7 +/- 0.9	
Burr diameter ||			0.903
1.25	20	7.8 +/- 0.9	
1.5	117	7.9 +/- 1.2	

> 1.5	35	7.7 +/- 1.1	
Ratio Burr diameter / Vessel diameter	170	0.156	0.042
Minimal speed used (rev/min)	170	-0.091	0.237
Maximal speed used (rev/min)	170	-0.078	0.316
Total number of burr runs	171	0.112	0.144
Total RA duration (seconds)	173	0.111	0.138

* Mean Ln(AUC 24h) +/- standard deviation.

† Spearman correlation coefficient.

‡ computed using the MDRD formula.

§ the smallest if several lesions treated using RA.

|| the largest if several burrs used.

ACS: Acute coronary syndrome. Cx: Left circumflex artery. GFR: Glomerular filtration rate. HDL: High density lipoprotein. LAD: Left anterior descending artery. LDL: Low density lipoprotein. LVEF: Left ventricular ejection fraction. PCI: Percutaneous coronary intervention. RA: Rotational atherectomy. RCA: Right coronary artery.

Online-only Supplementary Material S5 : Procedural and in-hospital outcomes (SAFETY Population, n=178)

	CLOPIDOGREL (n=88)			TICAGRELOR (n=90)			p-value
	n	%	CI 95%	n	%	CI 95%	
Death	1	1.1	0.0 – 3.3	0	0.0		0.494
Major Bleeding *	1	1.1	0.0 - 3.3	1	1.1	0.0 - 3.3	1.000
Minor Bleeding (BARC < 3)	4	4.5	0.2 - 8.8	3	3.3	0.0 - 7.0	0.719
Coronary dissection	1	1.1	0.0 - 3.3	6	6.7	1.5 - 11.9	0.118
Myocardial infarction	0	0.0	-	1	1.1	0.0 - 3.3	0.506
Ischemic stroke	1	1.1	0.0 - 3.3	0	0.0	-	0.494
Stent thrombosis	0	0.0	-	0	0.0	-	1.000

* BARC ≥ 3 or haemopericardium or coronary perforation.

CI : Confidence interval.
